# Supplementary material for: Learning curve and surgical outcome of robotic assisted colorectal surgery with ERAS program
Source: Sci Rep. 2022 Nov 29;12:20566. doi: 10.1038/s41598-022-24665-w (PMC9709162; doi:10.1038/s41598-022-24665-w)
Supplement: Supplementary file 2 — Supplementary Information 2. [file 41598_2022_24665_MOESM2_ESM.zip › RAL Raw data-Ñ[▒K/133/OP N_ñw╝╨░O▒Kñσ.pdf]

W92 / W92

臺中榮民總醫院
手術室護理記錄

[redacted]

基本資料

- 室別： 開刀房-17室，手術科別： CRS，手術類別： 常規，日期： 2021/12/15
- 進入時間： 0815 ； 麻醉開始時間： 0820 ； Time Out時間： 1001 ； 劃刀時間： 1003 ； 手術結束時間： 1520 送出時間： 1540 ；
- 麻醉方式： GE
  診斷： Malignant tumor of sigmoid colon 30 cm FAV
  手術名稱： Robot Assisted Colectomy(L)
  手術局部用藥： 2%Xylocaine jelly
  術前預防性抗生素：
    - 抗生素名稱一： Veterin 1GM IVD由麻醉護理人員執行
    - 時間： 0940
    - 上次簽章: 王昱瑀
  ，追加預防性抗生素：
    - 抗生素名稱一： Veterin 1GM IVD由麻醉護理人員執行
    - 時間： 1340
    - 上次簽章: 歐真勤
- 手術醫師： 林俊余，陳瑨夫，蔡濬樟，陳怡豪
- 麻醉醫師： VS 陳永銘
- 刷手護士A ： 洪千淳，時間： 0815，刷手護士B ： 歐真勤，時間： 0815，接替刷手一： 王昱瑀，時間： 1200，接替刷手二： 洪千淳，時間： 1230
  流動護士A ： 王昱瑀，時間： 0815，接替流動一： 歐真勤，時間： 1130，接替流動二： 邱任宏，時間： 1500
- 更多接替及流動護士： 接替刷手三： 廖子晴，時間： 1500

皮膚狀況

A-V Shunt： 無
術前： 完整
記錄者： 上次簽章: 王昱瑀
術後： 完整
記錄者： 上次簽章: 歐真勤

手術準備

手術臥式 單一手術臥式

-
    - 手術臥式： 截石臥式
    - 使用附件： 矽膠枕頭，腳架，其它： 廠商翻身用物
    - 皮膚準備： 10%Aq Betadine，Hibiscrub，2% Chlorhexidine
    - 電燒： 使用電燒: 電極片位置： 其它： 迴路電擊墊，雙極電燒，記錄者： 上次簽章: 王昱瑀

止血帶T： 無

植入物

無

標本送檢

- 細菌培養 無
- 細胞學 無
- 冰凍切片 無
- 病理標本
  組織名稱一： S colon
  ，
  組織名稱二： IMA root
  ，

組織名稱三： Distal cut end

- 截肢 無

標本交付 標本： 3 袋 , 標本： 0 盒 , 標本： 0 瓶 , 細胞學： 0 管 , Culture Tube： 0 管 , 被交付醫師： R 陳怡豪 ,

- 記錄者： 上次簽章: 邱任宏
- 時間： 1500

傷口術後敷料

傷口敷料： 傷口敷料： 無

- 請於圖上標示部位：

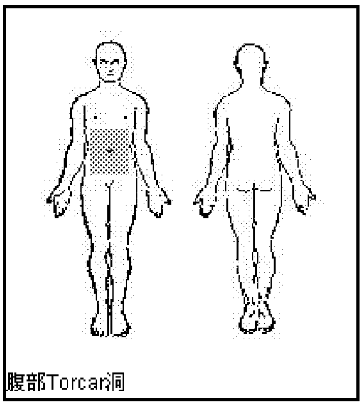

- 傷口敷料： menport

導管

導管	天數	導管資訊	插入日期	插入時間	更換日期	拔除日期	拔除時間	拔除原因	預計拔除/更換	預計拔除/更換日期
Epidural PCA(1)	1	部位:硬脊膜;	2021/12/15	0830						
Foley(1)	1	號碼:16 Fr.; 部位:膀胱;	2021/12/15	0900						
Sump NG Tube(1)	1	號碼:16 Fr.; 深度:50cm; 部位:胃;	2021/12/15	0930						
Double Lumen CVC(1)	1	號碼:8 Fr; 深度:15cm; 部位:jugular (右); 象限:RU;	2021/12/15	0905						
A-line (1)	1	號碼:20 G; 部位:左手;	2021/12/15	0900						

用物計數

- 手術中出血量： MINIMAL c.c.
- 手術中尿量： 1200 c.c.
-
  - 其它： 無

用物計數： 多切口同時縫合

- 1.器械： 正確 , 2.縫針： 正確 , 3.刀片： 正確 , 4.紗布： 正確 , 5.腹部墊： 未使用 , 6.紗球： 未使用 , 7.PEANUTS ： 未使用 , 8.紗條： 正確 , 9.絲帶： 未使用 , 10.針頭： 未使用 , 11.Vessel loop： 未使用 , 12.Boots： 未使用 , 13.Jelco： 未使用 , 14.棉條： 未使用 ,
  - 第一次：
    - 刷手護士 ： 洪千淳
    - 流動護士： 上次簽章: 歐真勤
    - 時間： 1420

,

  - 第二次：
    - 刷手護士 ： 洪千淳
    - 流動護士： 上次簽章: 歐真勤
    - 時間： 1430

,

  - 第三次：

- 刷手護士 ： 洪千淳
- 流動護士： 上次簽章: 歐真勤
- 時間： 1440

備註

備註： Davinci 0度 XI scopy:No.2

- 記錄者： 上次簽章: 王昱琄
- 時間： 1035

填寫護理人員 邱任宏

正式摘要

W92 / W92

doctorRank:B
